# Patterned Sensory Enhancement, a Music Rehabilitation Therapy for Elderly Patients with Neuromotor Deficit during the COVID-19 Pandemic: A Pilot Study

**DOI:** 10.3390/diseases12010011

**Published:** 2023-12-31

**Authors:** Iulia Toma, Anca Dinu, Ahmed Abu-Awwad, Mihai-Alexandru Sandesc, Simona-Alina Abu-Awwad, Razvan Nitu, Mihai Popean

**Affiliations:** 1 Faculty of Music and Theatre, West University of Timisoara, 300223 Timisoara, Romania; iulia.toma93@e-uvt.ro (I.T.); mihai.popean@e-uvt.ro (M.P.); 2 Department XVI—Medical Recovery, Victor Babes University of Medicine and Pharmacy, 300041 Timisoara, Romania; 3 Research Center for Assessment of Human Motion and Functionality and Disability, Victor Babes University of Medicine and Pharmacy, Eftimie Murgu Square, No. 2, 300041 Timisoara, Romania; 4 “Pius Brinzeu” Emergency Clinical County Hospital, Bld Liviu Rebreanu, No. 156, 300723 Timisoara, Romania; ahm.abuawwad@umft.ro (A.A.-A.); sandesc.mihai@umft.ro (M.-A.S.); alina.abuawwad@umft.ro (S.-A.A.-A.); nitu.dumitru@umft.ro (R.N.); 5 Department XV—Discipline of Orthopedics-Traumatology, Victor Babes University of Medicine and Pharmacy, Eftimie Murgu Square, No. 2, 300041 Timisoara, Romania; 6 Research Center Professor Doctor Teodor Șora, Victor Babes University of Medicine and Pharmacy, Eftimie Murgu Square, No. 2, 300041 Timisoara, Romania; 7 Department XII—Discipline of Obstetrics and Gynecology, Victor Babes University of Medicine and Pharmacy, Eftimie Murgu Square, No. 2, 300041 Timisoara, Romania

**Keywords:** neuromotor rehabilitation, musculoskeletal, relaxation, music therapy, elderly

## Abstract

**Simple Summary:**

The Patterned Sensory Enhancement (PSE) technique, a music-based rehabilitation strategy for neurological pathologies, is explored in this pilot study focusing on developing a music-drill protocol for elderly individuals with neuromuscular degenerative conditions. This study identifies specific music-composition parameters, including the key signature, rhythm, major scale, tonal system, movement, repetition, absence of movement, and use of musical instruments. This research suggests that collaboration between rehabilitation teams and musicians is crucial for optimizing the PSE technique, emphasizing the significance of tailored music stimuli in therapy. This study evaluates the therapy’s effectiveness using Takei dynamometer measurements before and after interventions. It concludes that personalized music compositions, aligned with individual patient preferences, are essential for optimal results in PSE therapy. The integration of patient-specific musical taste and recorded music during patient outcare is highlighted, underlining the need for ongoing collaboration between healthcare professionals and musicians to refine the PSE technique.

**Abstract:**

(1) Background: The Patterned Sensory Enhancement (PSE) technique refers to a music-based rehabilitation therapy strategy used in neurological pathologies. (2) Methods: This study aims at developing a music-drill protocol for recovery in elderly patients with neuromuscular degenerative pathologies. Each music drill is based on a number of specific music-composition parameters. The conclusions represent suggestions for further enhancing the PSE technique through collaborations between the rehabilitation team and the musicians involved due to the importance of the music stimuli used as a therapy. (3) Results: The music-composition parameters used in this study relate to the existence of music homogeneity factors such as the key signature and rhythm; the importance of the major scale and the tonal system in general; the obvious music suggestions of movement, repetition or absence of movement; the importance of rhythm; the introduction of musical instruments in order to harmonize the music material as much as possible; the connection between PSE music along with patients’ musical taste and the musical recording as a therapy during patient outcare; (4) Conclusions: The therapy efficiency was observed by measurements taken before and after the therapeutic intervention by means of a Takei dynamometer. The present study asserts that for a patient undergoing the PSE technique, the researcher should compose personalized music material adapted to each patient’s peculiarities.

## 1. Introduction

The ability to improve grip strength by integrating physical drills is a desirable therapy target in both healthy and disease-affected elderly patients [1] due to grip strength being a mortality predictor [2,3]. Moreover, elderly patients who concomitantly manifest low values of grip strength and BMI are at a higher risk of developing dementia [4].

General degeneration due to aging is a primary cause for muscle-mass decrease [5] resulting in gradually lower motor control and hand-function enactment [6], leading to a drop in grip strength. Furthermore, neuro-psychiatric pathologies lead to motor deficiencies, arm-grip strength decreasing in patients with stroke [7] and Parkinson’s disease [8,9]. Physical drills seem to deter degenerative processes (neuro-inflammation, vascularization, energy adjustment and homeostasis of the neurotrophic factors and of the neurotransmitters), which are causes for cognitive decline associated with aging, dementia, Parkinson’s and stroke [10].

Easy to medium-difficulty physical drills are maximally beneficial in chronic patients [10]. Moreover, structuring the drills in a series by gradually raising the target causes improvements in muscle strength based on training time duration [11]. 

The physical exercise accompanied by music stimulates force, tonus, agility, flexibility and self-control, also balancing cardiovascular homeostasis [12]. Music is relevant in neurorehabilitation, based on various areas of the human brain reacting to various musical factors [13,14]. Injured brains can respond to rhythm as a stimulus due to the fact that music and rhythm stimulate the repetition of motor movement [15]. 

Neurologic Music Therapy (NMT) “is a standardized system of clinical techniques that use the functional perception of all properties of music to train and retrain brain and behavior function” [15]. NMT is adapted to the patient’s needs and consists of 20 techniques (among them PSE) [16,17,18,19]. PSE is based on the principle that music motivates and illustrates the movement. PSE is part of the Neurologic Music Therapy (NMT) techniques and is relevant to patients’ disease management (rehabilitation, modulation and maintenance) [20,21,22,23]. All the elements of music (melody, harmony, rhythm, tempo, beat, dynamics, texture, timbre and pitch) are used to indicate spatial, strength, and temporal aspects of movement or fundamental motor patterns [23,24,25,26]. Through mirroring specific cues for single and discrete motions into functional movement sequences and patterns, PSE reflect operational activities of daily living [15,27,28,29,30].

In the present study, PSE was applied to patients with degenerative neuromotor pathologies in order to stimulate, via music, the cognitive and the motor systems that could result in modifying muscle strength. In order for the physical drills to run synchronously with the music, the patients required attention, limb coordination and stimulation of the musculoskeletal system. The results were evaluated by means of a grip dynamometer, a viable screening method for identifying elderly patients prone to disability [31], also indicative of muscle strength in post-stroke patients [32]. The dynamometer used was recommended by other studies as well [4,18,19], due to its measuring accuracy as far as muscle strength is concerned [20].

The authors of the present study believe that it is important to understand the general music-composition principles for a better outcome of the PSE therapy. Music in rehabilitation therapy (PSE) is a strong auditory stimulus for neuroplasticity [21,33,34,35]. In one of their studies, Bernatzky et al. encourage to find out which specific patterns or elements of music are leading to results that are beneficial to Parkinson’s patients [36]. The present article discusses PSE from a musical point of view. The hypothesis of this study was that muscle strength would increase by at least 5% as compared to the initial evaluation data. Cheong [15,37] recommends the use of material weights to be lifted and a 5% tempo increase during each drill session in order for the PSE to boost muscle force development, in addition to the improvement of the movement amplitude and rehabilitation.

## 2. Materials and Methods

A group of eight subjects with neuromotor pathologies (Alzheimer’s disease, Parkinson’s disease, and post-traumatic head injuries) agreed to participate in this research; after applying the exclusion criteria, six subjects qualified for the project (Table 1). Disease onset was 3 to 10 years before the present study.

Subjects showed:

- Presence of acute cardiac pathologies or of malignant cardiac arrhythmia.
- Presence of symptomatology or positive COVID-19 diagnosis.
- Inability to understand the researchers’ directions, or severe mental decline.
- Inability to maintain attention for the required periods of time.
- Presence of major auditory or visual affections preventing participation in the project.
- Pain felt during the experimental sessions.

New activities that imply regular physical training were discouraged. The patients were provided with a written consent form in order to participate in the experiment. The participants filled out a questionnaire regarding their age, weight, height, pathology, pathology onset, as well as patients’ musical preferences.

This research project took place in Timisoara at the Assistential Rehabilitation Centre for Persons at Risk. This study was part of a larger music-therapy protocol. Every session of this study took place 2 times/per week, for the duration of 4 weeks; each session started with a 15 min of PSE and ended with another 15 min of PSE. The drills were scheduled always at the same time (11–12 a.m.) and were monitored by a psychologist who helped with the questionnaires and a nurse who evaluated the subjects’ muscular strength. In order to adjust the initial study variables, two mock sessions were conducted, the first with a non-professional musician and the second with a group of experimental subjects.

Each stage of this study was run as per the protocol recommended by NMT [38]:

1. The patients performed the physical drills on the set tempo.
2. The physical exercises were carried out synchronously with the metronome.
3. The music was introduced.
4. The PSE exercises were carried out with no verbal directions, only keeping the music and the metronome.

In the present study, the music for PSE was composed by the authors (supervised by one of the authors that have a certification in NMT).

The music material used during the three PSE exercises was composed using the same key signature (C major) (the key signature suggests what key a section of music is composed in (it is a visual symbol) [39]) and the time signature (2/4) (the time signature indicates 2 numbers: the type of note which will receive one count and how many counts are in each measure of music [40]).

**Figure 1:**
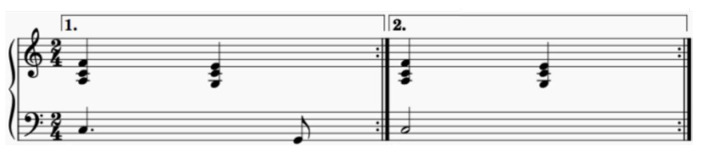
PSE no. 1.

**Figure 2:**
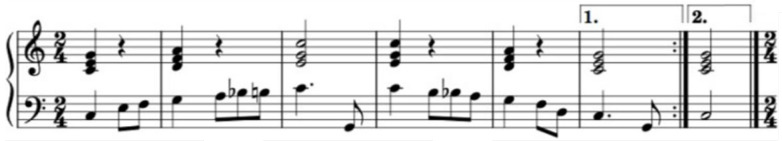
PSE no. 2.

**Figure 3:**
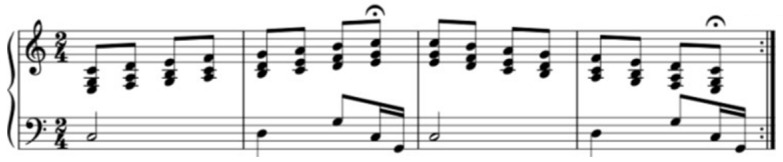
PSE no. 3.

The composition principles for the music material were:

- Drill no. 1 (Figure 1): was based on two alternative functions IV–I; the hand-gripping was synchronized with the subdominant chord (the Tonic (I) or subdominant (IV) chord refers to the name of the harmonic function (the name depends of the relationship to the home note/key) [41]) (beat 1); the later relaxation was synchronized with the second beat on the tonic chord (the tonic (I) or subdominant (IV) chord refers to the name of the harmonic function (the name depends of the relationship to the home note/key) [41]) (beat 2).

- Drill no. 2 (Figure 2) and no. 3 (Figure 3): an ascending melodic line was chosen during the arms’ upward movement, and a descending melodic line for the arms’ downward movement. The ascending and descending movements were synchronized with and followed the melody in the bass line, whereas the drills followed the constantly ascending and descending melodic line in the soprano in drill no. 3. Long note durations meant no movement but continuous grip.

During the three drills, the tonic (the tonic (I) and dominant (V) chord refers to the name of the harmonic function (the name depends of the relationship to the home note/key) [42]) chord in second inversion was used as an auditory stimulus to restart the movement on the music. The last bass note reminds of the dominant (the tonic (I) and dominant (V) chord refers to the name of the harmonic function (the name depends of the relationship to the home note/key) [43]) function of the scale. Thaut et al. recommend synchronizing the motor system with the auditory system using the basic rhythm structure that leads the pattern of movement [44].

Thus, rhythm structure was considered a priority in designing the movement and the music alongside factors such as register, dynamics, duration, harmony, tempo, music meter, rhythm pattern and music form.

The rhythm, in all the three drills, is based on equal note values that become increasingly shorter as the physical exercises increase in level of difficulty so as to also boost a number of bodily movements. In drill no. 1 (Figure 1), the rhythm was based on quarter notes, while the other two PSE exercises (Figure 2 and Figure 3) are based on eighth and sixteenth note values.

The triplet is associated with a chromatic melodic line in drill no. 2 is an example of stimulating the energy needed for the maximum lifting of the arms.

During both the mock and the testing sessions, an especially designated person was employed in order to perform the physical exercises in real time with the subjects. Although during later sessions this was no longer the case, this was necessary at the onset in order to help subjects familiarize with the protocol.

Cheong [15] ran a PSE-related study using an electronic piano, drawing on piano’s capacity to adapt rhythm and melody to various functions and activities. As an important note, our subjects did not respond well to the music being performed solely on the piano (third step). As a necessity, the protocol was slightly adjusted, and the examiner sung first the melodic line before introducing the piano. As a direct consequence, the subjects managed to better synchronize with the music.

During the mock session, the subjects reacted to the composed music different from what was expected. At the beginning, the subjects performed the physical exercises synchronously with the metronome; however, when the music was introduced, they stopped entirely and just listened to the music. Such a reaction may be due to subjects’ precarious contact with live music. Thus, a piano live performance sparked a reaction of increased attention and curiosity.

Different elements of music (tempo and melody) were also adjusted during the mock session. The first beat reference was a quarter note (q) = 50, which turned out to be too fast for the patients and was lowered to a q = 40. The music tempo (speed) was increased by 10% (q = 40 to 60), with the exception of one session when the tempo remained twice at the same metronome value of q = 50.

**Figure 4:**
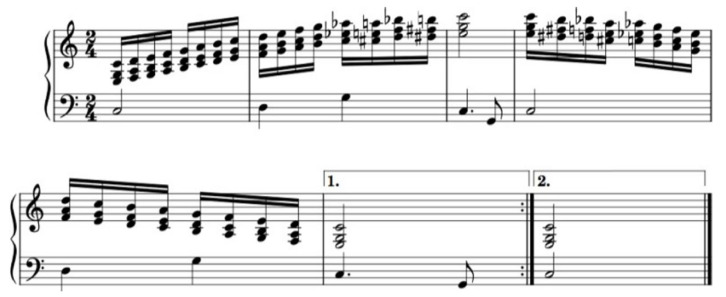
Initial PSE no. 3.

The melody was also adjusted for drill no. 3 (Figure 4), as the chromatic line was not well received by the subjects, the ascending and the descending melodic lines being longer than the duration needed for arm lifting and lowering. The melodic range was reduced to one octave while the rhythm was adjusted from 4/4 to 2/4, as the patients could not keep their arms lifted for 4 beats.

**Figure 5:**
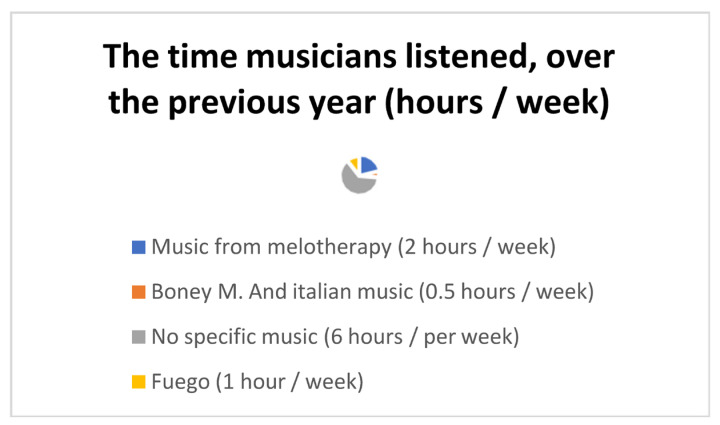
Questionnaire answers—Part I.

Initial delayed accommodation with the musical examples could further be explained by individual musical preferences in terms of style and genre as well as musical background. In order to assess the relation between music preference and background the subjects answered a set of questions at the beginning of this study. The questions addressed topics such as artist(s) listened to during the previous year; time dedicated to music listening (Figure 5), as well as favorite musician/band. The need for this questionnaire comes from the fact that, in many cases, music therapy sessions use random criteria for organizing playlists (later on will be discussed the importance of having principles of choosing the playlist for music therapy sessions). The favorite musician of the subject was: none (2 answers), Fuego (2 answers), Phil Collins (1 answer) and Liliac (1 answer).

During the first two weeks of the project, the music material was performed live by a pianist, whereas a recording was used in the last two weeks. Throughout the experiment, the subjects were verbally encouraged to maintain synchronicity during the physical drill. However, we do not recommend using recorded music for the initial rehabilitation therapy sessions since individual adjustments and elements of socialization may be necessary, especially for the elderly.

Bailey [24] found, in one of his studies, that post-live-music therapy, oncological patients reported lower muscle tension and levels of anxiety. He also found that live music also had a beneficial impact during the therapy sessions when patients exhibited increased physical response as compared to patients that used recorded music. Cheong [15] also recommends a similar protocol during PSE experiments. That being said, recorded music may still be very useful for the PSE technique during individual sessions the patients perform at home. This study took place in a room with regular chairs, allowing full range of motion. The devices used were a Takei T.K.K.5401 GRIP-D handgrip dynamometer (Takei Scientific Instruments Co., Ltd., Tokyo, Japan; accuracy 2 kg); a CASIO SA-76 electronic piano; an audio system optimally adjusted to the acoustic properties of the room (in terms of sound intensity and quality) and a metronome application installed on a smartphone (in order to obtain accurate synchronization between the musical rhythm and physical drills).

Each subject received two hand grips (LS3102B, 240 g weight, measuring 51 cm × 9.65 cm × 6.35 cm), made from safely grippable unpolished chrome steel. The hand grip devices increase hand, wrist and forearm strength [41], being also used in current medical rehabilitation strategies aimed at improving dexterity and lowering stress level [40,42,43]. In this study, the hand-grip used had a factory-set resistance at 10–20 kg [41].

The hand-grip drills were designed as follows: 3 drills, about 4 min each, for a total of 15 min, with a 1 to 2 min break between sessions [40,41,42,43,44]. The exercises were designed such that they apply an increasingly higher stress on muscle for the subjects to manage three different levels of difficulty. The subjects sat comfortably, eyes open, with both legs firmly set on the floor (soles in full contact with the floor) at a slightly open angle (approximately the abduction position). The default position at the beginning and the end of each drill was with both hands resting on the legs (each arm on the adjacent leg). The drills followed the rhythm of a particular music fragment out of three predefined choices.

- Drill no. 1: the subject held the hand-grips in both hands, in the default position. The drill consisted of gripping and relaxing the hands rhythmically, once per beat. The drill was followed by a 1 min break.
- Drill no. 2: the subject held the hand-grips in both hands, in the default position. This time, the drill movements were split into measures. During the first measure, the subject held the hands in the default position. During the second measure, the gripping and relaxing movements happened while the arms were lifted all the way ahead. During the third measure, the hands were raised all the way up and held there for a beat and a half with an eight-note short release at the end of the measure. The drill, then, followed the same in the opposite direction.
- Drill no. 3: the subject held the hand-grips in both hands, in the default position. During the first two measures, the hands were progressively lifted up in a continuous motion while the subject maintained full grip, followed by the opposite during the last two measures.

Alternative drills were prepared for special cases such as discomfort, fatigue, or pain. A research notebook was used in order to write down observations after each session. This study was preceded and followed by a muscle-strength assessment to observe any difference (if at all) in muscular performance. A prerequisite for these assessments was that subjects abstain from any intense physical exercise 24 h prior. These muscle-strength assessments were carried out during the same period of the day (11–12 a.m.), in a controlled environment. Following the protocol applied by Gatt et al. [28], the participants were standing, arms at their sides, with full elbow extension. There were a total of four measurements taken three times, with each hand, alternatively, with no rest periods between measurements. The peak value of each grip strength was noted, and an average was recorded. The statistical evaluation was carried out only to verify the effectiveness of the pilot project (later on, the project will be performed with a statistically significant group of subjects).

A short questionnaire (3 questions) designed to reveal the impact of music on the process of completing the physical exercises was filled out by the subjects after the experiment, 6 months later.

## 3. Results

The results are shown in Table 2. The percentage increase in muscle strength between the two evaluations is 9–94%. 71% of the subjects improved muscle strength with values above 30% higher than their initial values (results which should be replicated on a larger group of subjects).

In the post-experiment questionnaire, six months later, 100% of the subjects evaluated the music created especially for the experiment as boosting their feelings of well-being, playing a role in the increased relaxation of their musculoskeletal system, and supporting the completion of the drills.

## 4. Discussion

Peng [30] made an important effort to detail a number of PSE-technique music criteria; we believe that standardizing the general music parameters for the PSE-based rehabilitation therapy may prove beneficial for later studies. Examples include

- Using the same key signature/tonality and musical rhythm. The patients have a better memory recall when performing the physical exercises later. Due to the relationship between memory centers and music in the brain [41], a memory is generated by the movement correlated with the music. A major key as well as cadenzas (dominant-tonic or subdominant-tonic chords) to establish and stabilize the tonality are encouraged. Galińska [13] mentions the importance of using two music materials in the same key signature: both in C major. Later PSE studies may demonstrate a boosting and encouraging effect on the patients’ well-being by using the principle of similarity in tonality and musical rhythm as well as choosing major tonalities and the tonal system.
- Synchronicity between movement and music. The music supporting synchronous physical movement is important as even subjects without formal music education can perform the drills given that they receive proper stimulation within the music itself (specific chord-progressions and melodic lines). The PSE no. 1 (Figure 1) stimulated the hand gripping and relaxation by purposeful tension-release patterns during the harmonic progressions. The PSE no. 2 (Figure 2) stimulated hand gripping by imprinting an ascending-descending pattern to the melodic line, synchronized with the hands upward-downward motion. Galińska [12] and Cheong [15] also observed such correlation between the arm movement and melodic direction. The same principle was applied to the PSE 3 and 4.
- Distinct auditory stimulus. Subjects’ attention should be clearly prompted to the drill restart or the absence of movement using musical cues. In this study, the subjects recognized the dominant tone (the base of the tonic chord in second inversion) as signaling drill restart. The exercise ending or no movement was suggested by long-duration note values.
- Predictability. Generally speaking, it appears that a good balance between predictable and unpredictable musical elements might help subjects both better synchronize the physical movements with the rhythm and note values and also maintain interest. Predictability in music helps recognition of the compositional patterns, hence the triggering of neurotransmitter production able to generate good feelings, while unpredictability in music boosts curiosity and the wish to explore. Such mechanisms, which we believe are related to each subject’s musical taste, background, culture and education, and may prove beneficial in rehabilitation therapies.
- Prioritization of rhythm. During PSE therapy, it is of particular importance to emphasize the rhythmical aspects of the drills for both synchronicity and tempo. We noted the importance, for the subjects, of playing our music material at a high metronome level. However, a clear understanding of the relationship between tempo, rhythm pattern and arhythmical accent during simple and compound time signatures might play an important role in music playlist selection and/or PSE-music creation. Bukowska [31] mention the ability of the human brain to react to motion on a musical rhythm, even if the music is not the subject’s favorite. By prioritizing the metronome beat, our subjects managed to synchronize quite well with the musical rhythm composed especially for this study. However, we believe it important to make a clear distinction between rhythm pattern and musical rhythm as rhythm patterns are not necessarily part of a musical composition but can serve just as well for synchronicity purposes. Musical rhythm inherent in a musical composition is enhanced with melodic, chord or pitch information that can further assist with PSE-related drills.
- Usage of different musical instruments. It may be beneficial for PSE-related music to use different instruments in order to obtain different patient responses to harmony and timbre. For example, a harp could be used for its soft and warm timbre that invited relaxation, either as a solo instrument or in a group. Piano, for instance, could also be used in a solo group setting, with other string and percussion instruments such as cello, marimba, triangle, or tambourine. Different instruments naturally emphasize different timbre partials, making for a different auditory experience even when the same melodic line is used. Each instrumental timbre and sound particularities may be used in order to accommodate particular patient needs.

A state of mental and physical well-being as well as relaxation-boosting should be overarching targets in PSE therapy. Music material that is too simplistic, may have a negative impact on PSE-related patient response [12]. As a consequence, music creativity and ingenuity should apply in both, choice of musical instruments as well as the composition itself, by cooperation between the medical staff and musicians during both, research projects and rehabilitation therapy sessions. Galińska [12] further emphasizes the relevance of personalized music materials based on the peculiarities of the PSE therapy, even adjusting the PSE music to the patient’s needs in order to boost patient implication [15]. The relation between the music used in PSE-related therapy and patient implication/response was assessed in this study using the initial questionnaire. Generally, a lack of interest in regular music consumption is noted (Figure 5) among study participants. Such an observation normally affects both the playlist choices during the music therapy session as well as the music composed specifically for the PSE sessions. We encourage subjects to cultivate their taste in music, as the favorite music is shown to relax the human organism [32], boosting verbal and auditory memory as well as attention, thus shaping the patient’s emotional state [33]. Swallow and Sutton [34] state the importance of using relaxing, pleasant music for patients affected by Parkinson’s disease. We believe that the relaxation effect generated by music under PSE is due to the patients’ taste in music, as stimulated in the music therapy sessions. However, it is relevant to note the lack of scientific selection of the music playlist used in music therapy sessions (aspect not investigated in the present study). Later studies may investigate the parameters needed for the selection of the playlist for music therapy as well as the relation between the patients’ music culture and the PSE therapy effect. In the present study, the pathology of the subjects was also taken into consideration, particularly aspects related to memory recall.

The hypothesis was that muscle strength would increase by at least 5% as compared to the initial evaluation data. Even if the muscle strength increased by between 9 and 94%, research on larger groups of elderly patients with neuromotor deficit is further needed in order to validate the effectiveness of PSE-related therapy. In this study, the subjects were asked to perform the PSE-technique drills with their eyes open; by activating the ocular system, the level of sense stimuli is greater and the neuro-muscular control is improved [36,37]. In addition to the grip strength, the PSE technique boosted the subject’s attention capacity and arm coordination although, at this time, the latter was not the subject of this study. Also, the impact of the morning coffee on neuromotor activity could be further investigated in later studies: only one of the subjects had a cup of coffee per day and it is not clear how dietary choices may affect outcome.

The PSE rehabilitation protocol can be implemented outside of a research or specialized facility, home or in a video-conference format. Nevertheless, a one-on-one individual debut session with the patient as well as a later PSE-therapy customization session is necessary. Some researchers further recommend individualized physical exercises adapted to age and specific disabilities [11]. Cheong [15] applied PSE by creating 20 physical exercises for a total of 30 min. The authors of the current study found that, at least initially, for the elderly, the session should be split in two parts (i.e., 15 min–break–15 min). We also believe it beneficial that the therapy sessions be scheduled twice a week. Same frequency was used by other researchers in muscle strength rehabilitation therapy for the elderly [18]. The PSE technique was also applied to patients with stroke, in a once-a-week format, for a duration of 4 weeks; however, no objective assessment devices were used [15].

## 5. Conclusions

Music represents the main means for rehabilitation in the PSE technique. A number of composition parameters for PSE-related music composition were suggested, such as using the same tonality and musical rhythm, synchronicity between movement and music, distinct auditory stimulus, predictability, prioritization of rhythm, as well as usage of different musical instruments.

The initial hypothesis was confirmed—the subjects demonstrated muscle strength improvement significantly above the expected outcome. Larger studies may prove that the PSE technique is relevant for long-term rehabilitation therapy in elderly patients with neuromotor challenges.

## Figures and Tables

**Table 1:** Demographical Data.

Gender	4 Males
	2 Females
Age	73.8 Mean
BMI	
Subject 1	21.13
Subject 2	24.22
Subject 3	25.51
Subject 4	22.22
Subject 5	26.63
Subject 6	26.56

**Table 2:** Results.

Subjects	Initial Evaluation	Final Evaluation
Subject 1	17.9 kg	34.7 kg
Subject 2	7.9 kg	11.1 kg
Subject 3	8.4 kg	13.8 kg
Subject 4	20.1 kg	28.2 kg
Subject 5	33.7 kg	36.6 kg
Subject 6	24.7 kg	32.0 kg

## Data Availability

The data you is available with the corresponding author of the study. You may contact the corresponding author for further details and access to the relevant data. Additionally, a copy of the data is also stored in our hospital’s records. If you require access to the data from our hospital, please feel free to reach out to our data management department.
